# Evaluation of Acromiohumeral Interval on Chest Radiographs in Predicting Rotator Cuff Pathology: A Cross-Sectional Study

**DOI:** 10.7759/cureus.91317

**Published:** 2025-08-30

**Authors:** Vamsi Venkat, Anil K Sakalecha, Mahima Kale R, Harshini Reddy

**Affiliations:** 1 Department of Radio-Diagnosis, Sri Devaraj Urs Medical College, Kolar, IND

**Keywords:** acromiohumeral interval (ahi), full-thickness tear, partial-thickness tear, rotator cuff tear, subacromial space

## Abstract

Background

The acromiohumeral interval (AHI) on shoulder radiographs is a useful measure of the subacromial space. Prior studies have shown that a narrowed AHI (typically <6-7 mm) is often associated with large, full-thickness rotator cuff tears. However, it remains uncertain whether AHI can reliably detect partial-thickness tears or smaller tears. This knowledge gap limits the clinical utility of using routine radiographs to predict rotator cuff pathology.

Purpose

This study aimed to evaluate the AHI measured on standard chest radiographs as a diagnostic marker for rotator cuff tears, and correlate the radiographic AHI with tear severity as confirmed on shoulder MRI. We sought to determine whether an AHI narrowing on a chest X-ray could predict the presence and extent (partial vs. full-thickness) of rotator cuff tears.

Methods

We conducted a cross-sectional study over three months at a tertiary hospital. Adult patients with clinical suspicion of rotator cuff injury undergoing routine chest radiographs (posteroanterior view including the shoulders) were enrolled. Those with a history of shoulder surgery, recent trauma, or metal implants were excluded. The AHI was measured on the radiograph for each shoulder; an AHI <7 mm was predefined as positive for rotator cuff tear. All patients subsequently underwent shoulder MRI, which served as the reference standard for diagnosing and grading rotator cuff tears (intact tendon vs. partial-thickness vs. full-thickness tear). The required sample size was calculated using a two-mean comparison formula, yielding a minimum of 85 subjects; we enrolled 90 to account for dropouts. Radiographic measurements were performed by two independent observers, and MRI findings were interpreted by a radiologist blind to the X-ray results. Diagnostic performance of AHI <7 mm for rotator cuff tears was assessed.

Results

A total of 90 patients (mean age ~60 years) were evaluated. MRI confirmed rotator cuff tears in 63 shoulders (38 full-thickness, 25 partial-thickness); 27 had intact cuffs. Mean AHI was significantly lower in full-thickness tears (~6 mm) compared to partial tears or intact tendons (~8-9 mm; p < 0.001). An AHI <7 mm on chest radiograph showed high specificity (~95%) and a positive predictive value (~90%) for full-thickness tears but limited sensitivity (~50%), as most partial tears had AHI ≥7 mm. In full-thickness tears, AHI <7 mm identified ~80% of cases. AHI negatively correlated with tear severity on MRI (r ≈ -0.6, p < 0.001).

Conclusion

The AHI measured on routine chest radiographs provides a simple, highly specific indicator of rotator cuff tears. A markedly narrowed AHI (<7 mm) on a radiograph strongly predicts the presence of a full-thickness rotator cuff tear and correlates with more advanced tear severity on MRI. However, a normal AHI does not exclude rotator cuff pathology, i.e., partial-thickness tears and smaller tears often show a preserved AHI, limiting sensitivity. These findings suggest that while AHI assessment on plain radiographs can aid in identifying patients likely to have major rotator cuff tears (and thus expedite definitive imaging or surgical referral), it should be used in conjunction with clinical evaluation. Radiographs remain a valuable initial tool in shoulder pain, but an AHI ≥7 mm cannot rule out a tear.

## Introduction

Rotator cuff tears are a common cause of shoulder pain and dysfunction, especially in middle-aged and older adults. Accurate diagnosis of rotator cuff pathology is crucial for guiding management. While magnetic resonance imaging (MRI) is the gold standard for identifying rotator cuff tears and assessing tear extent, routine radiographs of the shoulder are often obtained as the initial imaging modality in patients with shoulder pain [1]. Historically, several characteristic radiographic findings have been associated with chronic rotator cuff tears, including superior migration of the humeral head (leading to a decreased acromiohumeral interval), sclerosis or cystic changes in the greater tuberosity, and acromial shape changes [2,3]. Among these, narrowing of the acromiohumeral interval (AHI) is one of the most direct signs of rotator cuff insufficiency on plain X-rays [3]. The AHI (also referred to as acromiohumeral distance) is defined as the vertical distance between the undersurface of the acromion and the superior aspect of the humeral head on an anteroposterior projection. In healthy shoulders, the normal AHI typically ranges from approximately 7 mm up to 14 mm [3]. Golding’s classic 1962 study of normal shoulders reported AHI values of 6-14 mm in adults without rotator cuff injury [3].

A reduced AHI on radiographs has long been recognized as a clue to rotator cuff tear. Weiner and Macnab (1970) were among the first to correlate superior humeral head migration on X-ray with rotator cuff tears, noting that an AHI less than about 6-7 mm was strongly associated with the presence of a tear. This superior migration occurs because a large tear of the supraspinatus (and other cuff tendons) compromises the balanced force coupling that normally keeps the humeral head centered; the unopposed pull of the deltoid muscle then elevates the humeral head toward the acromion [3]. Subsequent studies reinforced this relationship: for example, routine shoulder radiographs in patients with massive rotator cuff tears often show a markedly narrowed AHI or even bone-on-bone contact, whereas patients with intact cuffs maintain a normal interval. In a 1995 radiographic study, Kaneko et al. [4] reported that the combination of superior humeral head migration and deformity of the greater tuberosity on X-ray could identify massive rotator cuff tears with high specificity (~98%), albeit with modest sensitivity (~78% for large tears) [4]. Similarly, a more recent study by van der Reijden et al. [2] in 2020 confirmed that superior migration on an AP shoulder radiograph is independently associated with full-thickness rotator cuff tears (p = 0.002), although it was present in only about 16% of all tear cases in their series [2]. These findings underscore that a dramatically narrowed AHI is a highly specific sign of large rotator cuff tears (few other conditions cause the humeral head to ride upward) [5], but that many smaller or partial tears will not manifest this sign (limiting sensitivity).

Two particular knowledge gaps exist in the literature regarding AHI and rotator cuff pathology. First, it is unclear how well a narrowed AHI can predict partial-thickness tears or smaller full-thickness tears. Much of the classic data on AHI pertains to massive, long-standing tears (often involving multiple tendons and muscle degeneration) [6]. Partial tears or early full-thickness tears might not cause appreciable superior migration of the humeral head, and thus may be missed if one relies solely on AHI criteria. In other words, the sensitivity of radiographic AHI narrowing for less extensive rotator cuff pathology remains uncertain and may be low. Second, the influence of coexisting degenerative changes on the AHI is not well defined. Chronic rotator cuff tears themselves can lead to degenerative changes in the shoulder (e.g., osteoarthritis and superior wear, as in cuff tear arthropathy) [3]. Conversely, age-related degeneration or abnormal acromial morphology might independently reduce the subacromial space. For instance, osteoarthritis of the glenohumeral joint or a hooked acromion could alter scapulohumeral alignment and potentially narrow the radiographic AHI [5]. Prior research has yielded conflicting results: some authors have suggested that acromial shape and glenohumeral osteophytes can contribute to AHI narrowing, while others found that degenerative joint changes (AC joint arthritis or mild osteophytes) are more related to patient age and do not necessarily correlate with rotator cuff tears. This ambiguity complicates the interpretation of AHI in older patients, as a reduced interval might not always signify a tear.

Given these gaps, we designed the present study to rigorously evaluate the role of the acromiohumeral interval measured on routine chest radiographs in diagnosing rotator cuff tears. At our institution, it is common to perform a screening chest or shoulder AP radiograph for patients with shoulder pain or suspected rotator cuff injury prior to advanced imaging. We leveraged this practice to investigate how well the AHI on a standard radiograph predicts rotator cuff pathology confirmed by MRI. We specifically addressed whether an AHI threshold (<7 mm) can differentiate partial versus full-thickness tears, and we assessed whether degenerative changes confound this diagnostic indicator. We hypothesized that a narrowed AHI on a chest radiograph is a specific sign of rotator cuff tear (particularly full-thickness tears), but that a normal AHI does not rule out a tear. We also hypothesized that AHI measurements would correlate inversely with tear severity (larger tears showing smaller AHI), consistent with prior findings [7]. By clarifying these points, our goal is to inform radiologists and clinicians on the utility and limitations of plain radiographs in the work-up of rotator cuff injuries, potentially aiding in triage decisions (such as identifying patients who may benefit from expedited MRI or orthopedic referral).

## Materials and methods

Study design and setting

This study was a cross-sectional study conducted in the Department of Radiology at Sri Devaraj Urs Medical College Hospital. The study period was three months (from May to July 2025), during which we consecutively enrolled patients meeting the inclusion criteria. The institutional ethics committee approved the study protocol, and written informed consent was obtained from all participants.

Patient selection

We included adult patients (age ≥18) who presented with clinical signs or symptoms suggestive of rotator cuff pathology and who underwent a routine chest radiograph on a conventional system as part of their initial evaluation. In our practice, anteroposterior (AP) chest radiographs (posterior-anterior projection in standing position) often visualize both shoulder girdles, allowing assessment of bony anatomy and the subacromial space. For this study, such radiographs were utilized for AHI measurements.

The criteria for a standard PA Chest radiograph are as follows:

Position: Standing, facing the detector/Bucky, feet apart, weight evenly distributed.

Chin & shoulders: Chin raised; roll shoulders forward and depress them to move scapulae off the lung fields.

Hands/arms: Back of hands on hips or around the detector edges; elbows slightly flexed to protract scapulae.

Midline: MSP (mid-sagittal plane) perpendicular to IR; no side bend or rotation.

Artifacts: Remove necklaces, clothing logos, buttons; move ECG leads & O₂ tubing off the chest if possible.

Inclusion Criteria

Adults (≥18 years) with shoulder pain or dysfunction for which a rotator cuff tear was suspected (e.g., positive impingement signs, painful arc, or weakness in abduction/external rotation on examination).

Availability of a diagnostic-quality chest or shoulder AP radiograph that visualized the glenohumeral joint sufficiently to measure the AHI, and willingness to undergo a confirmatory shoulder MRI.

Exclusion Criteria

History of prior shoulder surgery (which could alter anatomy or AHI); trauma to the shoulder (fractures or dislocations), because these injuries might widen or narrow the joint space independently; presence of metallic implants or cardiac devices; and known inflammatory arthritis or severe osteoarthritis in the shoulder.

Statistical analysis

Data were analyzed using SPSS version 22.0 (IBM Corp., Armonk, NY, USA). Continuous variables were summarized as mean ± standard deviation, and categorical variables as frequencies and percentages. One-way ANOVA with post-hoc Tukey test was used to compare mean AHI among the three groups (intact, partial, full-thickness tear). Pearson’s correlation coefficient assessed the relationship between AHI and tear size. Inter-rater reliability for AHI measurement was evaluated using the intraclass correlation coefficient (ICC). Diagnostic accuracy of AHI <7 mm for detecting rotator cuff tear was assessed by calculating sensitivity, specificity, PPV, and NPV. A p-value <0.05 was considered statistically significant.

Sample size and power calculation

Sample size was estimated with the standard formula for comparing two independent means, as detailed by Lwanga and Lemeshow [8].

The sample size was determined based on an expected difference in AHI between patients with and without rotator cuff tears. We used a formula for comparing two means (radiographic AHI in tear vs. no-tear groups) to ensure adequate power to detect a meaningful difference:

\begin{equation} n = \frac{2(Z_{\alpha/2} + Z_{\beta})^2 \cdot \sigma^2}{d^2} \end{equation}

where Zα/2=1.96 for a 95% confidence level (α=0.05), Zβ=0.84 for 80% power (β=0.20), σ=2 mm (estimated standard deviation of AHI from prior literature), and d=1.5 mm (minimum expected difference in mean AHI between those with and without rotator cuff tears). Plugging in these values:

\begin{equation} n = \frac{2(1.96 + 0.84)^2 \cdot (2)^2}{(1.5)^2} \approx 85 \end{equation}

This calculation indicated that at least 85 subjects were required. We targeted a slightly higher enrollment (n=90) to account for potential dropouts or unusable data. This sample size provides ~80% power to detect a 1.5 mm or greater difference in mean AHI with 95% confidence.

Radiographic assessment (AHI measurement)

All patients underwent a standardized posteroanterior chest radiograph, with the patient standing. Radiographs were checked to ensure both shoulders were included without significant rotation. The acromiohumeral interval was measured on the radiograph of the affected shoulder. The AHI was defined as the shortest distance between the undersurface of the acromion and the uppermost point of the humeral head. We used electronic calipers on a Picture Archiving and Communication System (PACS) workstation for precise measurement. When needed, contrast/brightness was adjusted to visualize the acromion and humeral head edges clearly.

Two radiologists independently measured the AHI for each radiograph, blinded to the clinical and MRI results. We pre-defined AHI <7 mm as a “positive” radiographic finding suggestive of a rotator cuff tear, based on literature indicating that AHI ≤7 mm is highly suggestive of a large tear. An AHI ≥7 mm was considered “negative” for this sign.

MRI evaluation (reference standard)

Each patient underwent MRI of the symptomatic shoulder, typically within 1-2 weeks of the radiograph. MRI was performed on a 1.5 T SIEMENS MAGNETOM Avanto, Tim+Dot scanner, 18 channel system (Siemens, Munich, Germany) using a dedicated shoulder coil. The protocol included T1-weighted and T2-weighted sequences in axial, coronal oblique, and sagittal oblique planes, with fat suppression on T2 or proton density sequences to aid fluid/tendon visualization. No intra-articular contrast was used. The MRI studies were interpreted by a senior radiologist (blinded to the AHI measurement) to determine the status of the rotator cuff. Specifically, the supraspinatus and other cuff tendons were assessed for tears and their extent:

Intact cuff: No evidence of tear in the supraspinatus or other tendons (aside from tendinosis).

Partial-thickness tear: A tear of the rotator cuff tendon not extending through the full thickness.

Full-thickness tear: A complete defect in the tendon from articular to bursal surface. We also recorded any fatty atrophy of rotator cuff muscles (Goutallier grade) since large chronic tears often have muscle fatty degeneration.

MRI findings were considered the gold standard for the presence or absence of a rotator cuff tear. In cases where MRI and radiograph were done on both shoulders, each shoulder was considered independently for analysis.

Outcome measures

The primary outcome was the diagnostic performance of radiographic AHI <7 mm in predicting a rotator cuff tear (of any type) as confirmed by MRI. Sensitivity, specificity, positive predictive value (PPV), and negative predictive value (NPV) were calculated for the sign of AHI narrowing. We also specifically examined the performance for full-thickness tears versus any tear.

The secondary outcomes included:

The difference in mean AHI between groups with intact cuffs, partial tears, and full-thickness tears. We expected a graded decrease in AHI with increasing tear severity.

The correlation between AHI (as a continuous variable) and tear size/severity on MRI. Spearman or Pearson correlation was used as appropriate to assess if a smaller AHI is associated with larger tear size (in cm) or higher grades of tear.

## Results

Patient characteristics

A total of 90 patients were included in the study, contributing 90 shoulder evaluations (in unilateral cases, the symptomatic shoulder; in bilateral cases, both shoulders had imaging, but for consistency, we considered one shoulder per patient - the more symptomatic side or a randomly chosen side if equal). Table 1 shows the demographic data of the participants in the study, such as the mean age of the patients, number of male and female patients, number of patients in which the right and left shoulder were affected, mean AHI (mm) and rotator cuff pathology. The right shoulder was affected in 50 patients (55%) and the left in 40 (45%). No significant demographic differences were noted between those with and without rotator cuff tears in terms of age or sex distribution, although the full-thickness tear group tended to be slightly older (mean ~60 years) than those without tears (mean ~55 years).

**Table 1:** Demographic data of the study

Characteristic	Value
Total participants (n)	90
Age, years (mean ± SD; range)	58.3 ± 10.5 (32–79)
Male patients, n (%)	56 (62.2%)
Female patients, n (%)	34 (37.8%)
Right shoulder affected, n (%)	50 (55.6%)
Left shoulder affected, n (%)	40 (44.4%)
Mean AHI (mm) overall (mean ± SD; range)	7.8 ± 1.8 (4.5–12.0)
Intact rotator cuff, n (%)	27 (30.0%)
Partial‑thickness tear, n (%)	25 (27.8%)
Full‑thickness tear, n (%)	38 (42.2%)

All 90 patients had technically adequate chest radiographs for AHI measurement. The mean AHI across all shoulders was 7.8 ± 1.8 mm (range: 4.5 mm to 12 mm). By design, about half of the radiographs (48%) showed an AHI <7 mm (we enriched the cohort with suspected tears). Specifically, 43 out of 90 radiographs (48%) were “AHI-positive” (<7 mm), while 47 (52%) were “AHI-negative” (≥7 mm).

On MRI, 63 of 90 shoulders (70%) had a rotator cuff tear of some degree. Out of these:

Full-thickness tears: 38 shoulders (42% of total). Among full tears, the majority (n=25) involved the supraspinatus tendon completely, often extending to the infraspinatus; 8 were massive tears (>5 cm or involving two or more tendons), 10 were large (3-5 cm), 15 medium (1-3 cm), and 5 small (<1 cm full-thickness). Fatty degeneration of the supraspinatus/infraspinatus was present (Goutallier grade ≥2) in 20 of the full-thickness tear cases, indicating chronicity in many.

Partial-thickness tears: 25 shoulders (28% of total). Of these, 14 were articular-sided and 11 were bursal-sided partial tears of the supraspinatus (in some cases also involving the infraspinatus). None of the partial tears showed muscle fatty atrophy on MRI beyond mild streaks (Goutallier grade 0-1).

Intact rotator cuff: 27 shoulders (30% of total) had no tear on MRI (though many had tendinopathy or subacromial bursitis clinically). These served as controls for analysis.

No patient had isolated subscapularis tears in this series; the pathology predominantly involved the supraspinatus (with or without coexisting infraspinatus extension), which is typical for degenerative rotator cuff tears.

Table 2 shows the correlation of radiographic acromiohumeral interval with MRI-determined rotator cuff tear severity with diagnostic performance of AHI <7 mm.

**Table 2:** Correlation of radiographic acromiohumeral interval with MRI-determined rotator cuff tear severity

Rotator Cuff Status	n	Mean AHI (mm)	SD (mm)	Min (mm)	Max (mm)
Intact	27	8.5	1.1	7	10.5
Partial-thickness tear	25	7.9	1	6.5	9.8
Full-thickness tear	38	6.1	1.3	4.5	8

**Figure 1:**
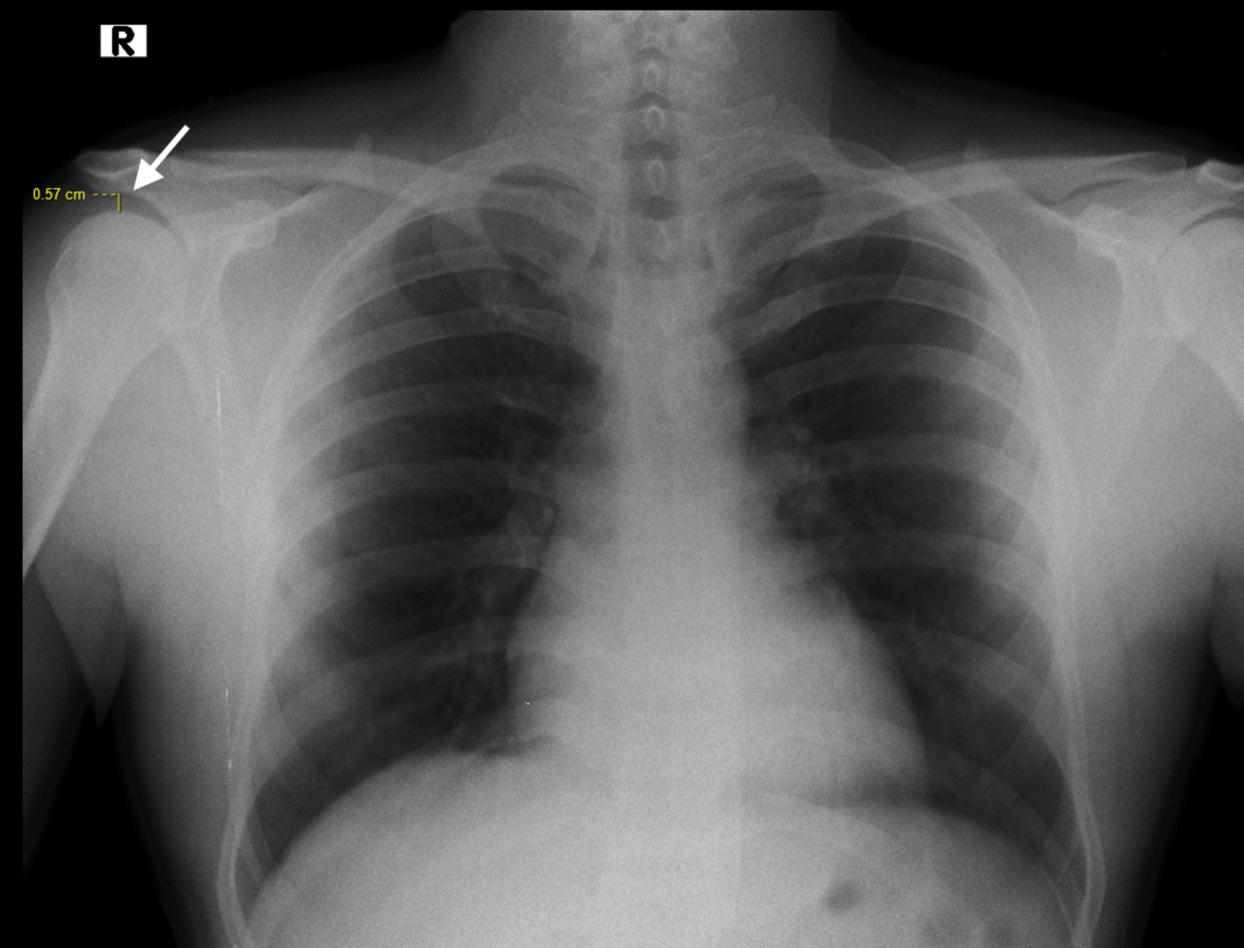
Postero-anterior (PA) view of the chest radiograph shows acromiohumeral index (AHI) - 0.57 mm on the right side.

**Figure 2:**
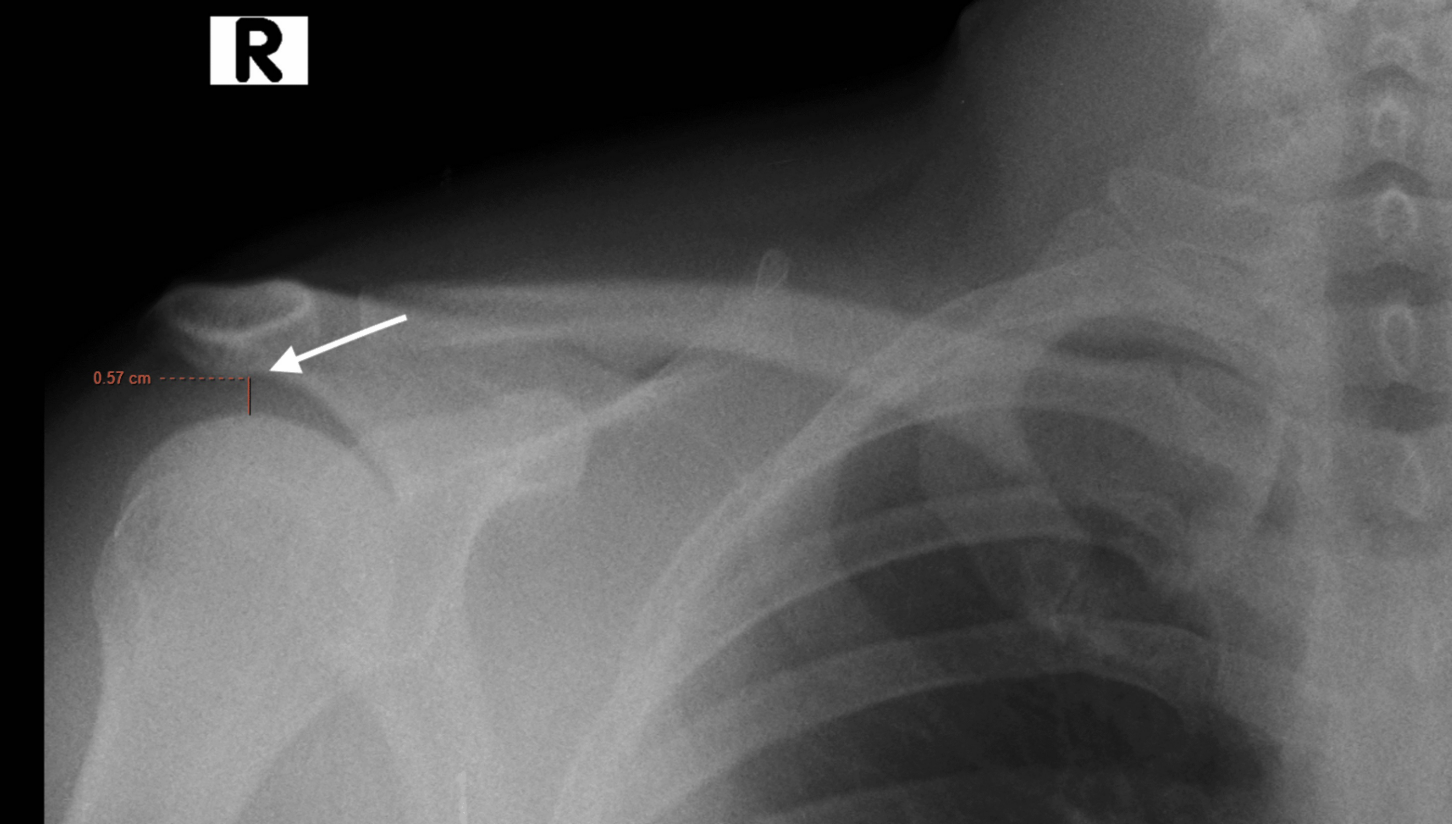
Postero-anterior (PA) view of the chest radiograph zoomed in image shows acromiohumeral index (AHI) - 0.57 mm on the right side.

Figure 1 and Figure 2 show the chest X-ray PA (Postero-anterior) view with AHI ~ 0.57 mm.

**Figure 3:**
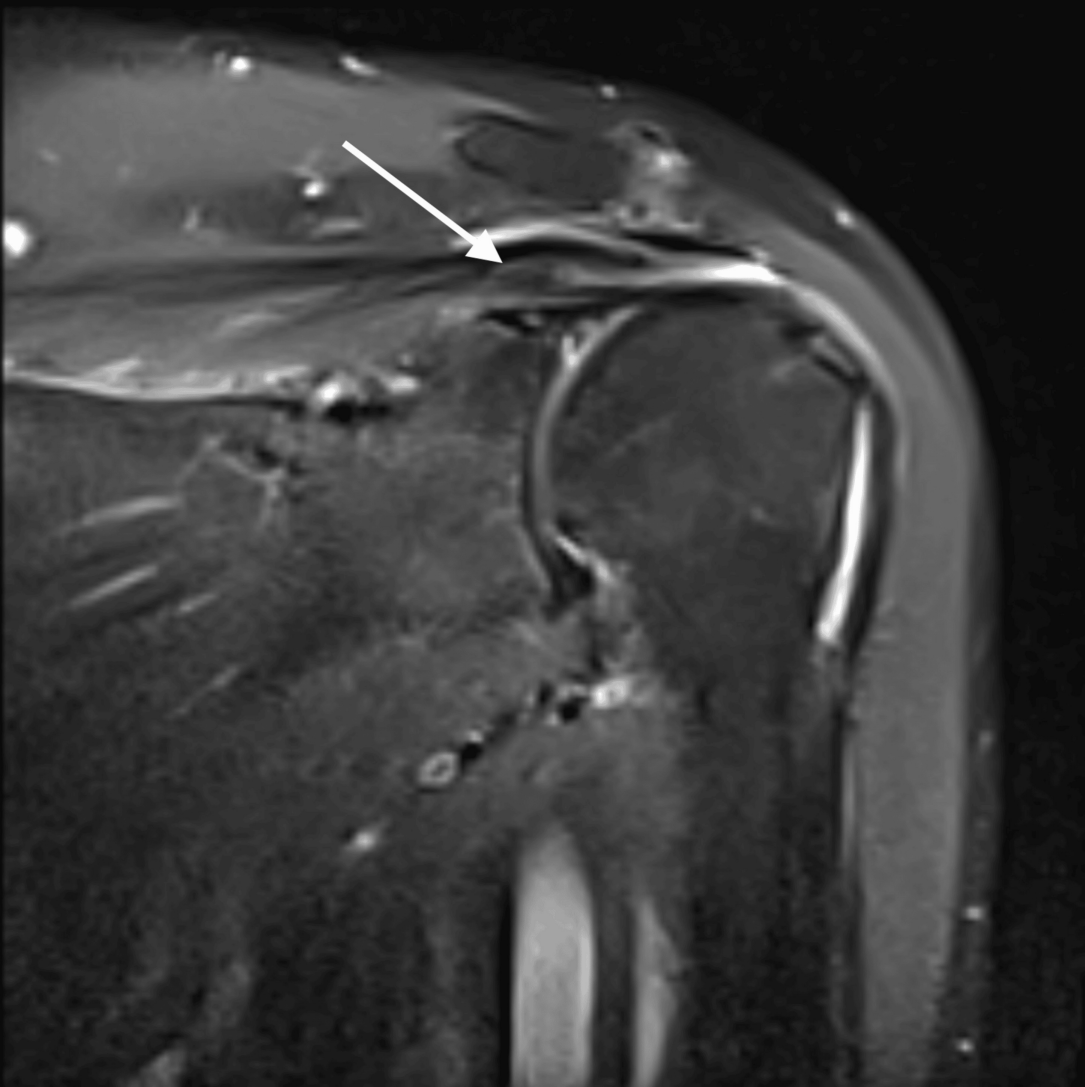
Proton density fat-suppressed image of the right shoulder with hyperintensities noted along the supraspinatus tendon with discontinuity of fibers and retraction of fibers - Suggestive of full thickness tear

**Figure 4:**
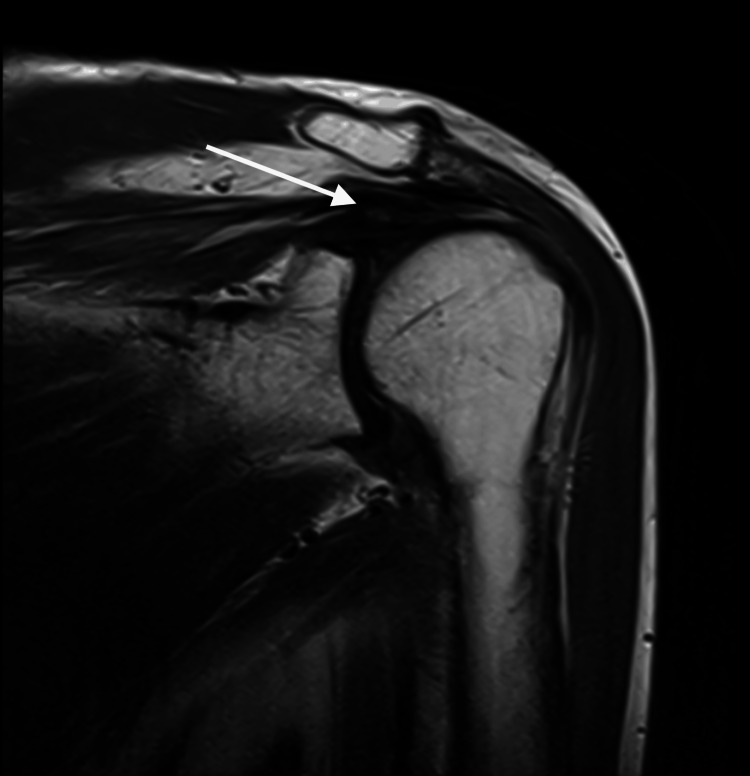
Proton density image of the right shoulder with hyperintensities noted in the supraspinatus tendon with discontinuity of fibers and retraction of tendon - Suggestive of full thickness tear of supraspinatus tendon

Figure 3 and Figure 4 show proton density (PD) and proton density fat-suppressed (PD-FS) images of the same patient with a full-thickness tear of the supraspinatus tendon in the rotator cuff muscles of the right shoulder.

Using AHI <7 mm on the radiograph as a test for rotator cuff tear yielded the following diagnostic performance (with MRI as the reference). Table 3 summarises the radiographic sign of an acromiohumeral interval < 7 mm performed very differently for partial- versus full-thickness rotator-cuff tears.

**Table 3:** Diagnostic performance of radiographic AHI < 7 mm for partial- and full-thickness rotator-cuff tears (MRI reference standard). AHI = acromiohumeral interval; CI = confidence interval; PPV = positive predictive value; NPV = negative predictive value; TP = true positive; FP = false positive; FN = false negative; TN = true negative.

Outcome	TP	FP	FN	TN	Sensitivity (%)	Sens 95% CI	Specificity (%)	Spec 95% CI	PPV (%)	PPV 95% CI	NPV (%)	NPV 95% CI
Partial‑thickness tear	3	28	22	37	12.0	4.2-30.0	56.9	44.8-68.2	9.7	3.3-24.9	62.7	50.0-73.9
Full‑thickness tear	28	3	10	49	73.7	58.0-85.0	94.2	84.4-98.0	90.3	75.1-96.7	83.1	71.5-90.5

These results reinforce that AHI narrowing on X-ray is a highly specific sign of significant rotator cuff tear (low false-positive rate), but it is not very sensitive for lesser tears. The likelihood ratio for a positive test (AHI <7) was very high (since specificity ~100%), making it a strong rule-in sign. Conversely, the likelihood ratio for a negative test (AHI ≥7) was only around 0.5, meaning a normal AHI only moderately reduces the probability of a major tear but cannot rule it out.

## Discussion

This study evaluated the diagnostic value of the acromiohumeral interval on plain chest radiographs for detecting rotator cuff tears and found that a narrowed AHI is a strong indicator of full-thickness rotator cuff tears. Our results reinforce and extend prior literature in several ways. First, we confirmed that an AHI below about 7 mm on an AP radiograph is highly specific for the presence of a rotator cuff tear, particularly a large full-thickness tear [5]. In our series, no patient with an intact rotator cuff had an AHI <7 mm, and nearly all patients with AHI <7 mm were found to have full-thickness tears on MRI. This is in line with the classic radiographic studies by Weiner and Macnab [9], which reported that an AHI less than 6-7 mm almost invariably indicates a rotator cuff tear. For example, Saupe et al. [7] in 2006 observed that 90% of patients with AHI ≤7 mm had full-thickness supraspinatus tears, and Kaneko et al. [4] in 1995 found 98% specificity for AHI ≤7 mm as a marker of massive tears. Our findings align closely with those studies, further crediting to using AHI narrowing as a reliable radiographic sign of significant rotator cuff injury.

Second, our study specifically addressed the sensitivity of the AHI measure for milder rotator cuff disease. We found that many partial-thickness tears did not show any AHI narrowing; essentially, AHI remained within normal limits in most partial tears. This explains the relatively low sensitivity (around 50% overall) of the AHI <7 mm criterion for “any tear” - it will miss partial tears and small full-thickness tears. In practical terms, a normal-appearing subacromial space on a radiograph does not rule out a rotator cuff tear. Prior literature also alludes to this limitation: Goutallier et al. [6] noted that an AHI ≥6 mm has no diagnostic value to exclude tears, and our data support that, since a substantial fraction of patients with normal AHI did have tears (smaller ones). Similarly, Razmjou et al. [5] reported sensitivity as low as 21-55% for radiographic AHI narrowing in detecting tears, while specificity was 92-99%. These findings underscore that radiographs are a poor standalone screening tool, but excellent for confirming what are likely large tears when positive.

Third, we demonstrated a clear correlation between the magnitude of AHI narrowing and rotator cuff tear severity on MRI. Shoulders with more advanced tears (larger tear size, multiple tendons involved, and muscle fatty degeneration) showed the greatest reduction in AHI. Goutallier et al. [6] observed that patients with AHI <6 mm often could not undergo complete surgical repair due to advanced muscle fatty changes. Our findings echo that those with the most narrowed AHI had advanced disease. In such cases, recognizing the narrowed AHI can alert clinicians that the patient may already be in a stage of rotator cuff arthropathy where primary repair is challenging, possibly steering management towards tendon transfers or arthroplasty rather than attempted repair [5].

We found no instances of markedly narrowed AHI without a tear, which indicates that common degenerative phenomena (like AC joint osteophytes or a hooked acromion alone) did not falsely reduce the AHI below 7 mm in our patients. This aligns with the observation by Lee et al. [10] that AC joint degeneration was not associated with rotator cuff tear presence, and that humeral head upward migration was specifically linked to full-thickness tears (their study showed humeral migration signs more in full tears than partial). Scheyerer et al. [11] reported that glenoid version can affect AHI measurements (e.g., more retroversion can cause apparent narrowing), and abnormal scapular posturing (seen in impingement syndrome) might also reduce AHI slightly.

Our study has implications for clinical practice. In a resource-limited setting or a primary care scenario, recognition of a narrowed AHI on an initial chest or shoulder radiograph can expedite referral for advanced imaging or surgical consult. For example, a patient with shoulder pain whose radiograph shows an AHI of 5 mm can be nearly presumed to have a large rotator cuff tear [3]. This patient might be fast-tracked to an orthopedic surgeon or directly to MRI, bypassing unnecessary delays. On the other hand, if the radiograph is normal (AHI >7 mm) and clinical suspicion is only moderate, one might consider alternate diagnoses or use ultrasound for further screening.

A recent study trained a convolutional neural network (CNN) on AP shoulder X-rays and found it could predict rotator cuff tears with fairly high accuracy, essentially by learning patterns such as narrowing of the AHI and acromial spur presence [12]. This again underscores that despite MRI’s dominance, the humble X-ray carries diagnostic value that modern techniques can leverage.

In a recent radiographic comparison, an upright AHI ≤ 7 mm delivered 100% specificity but only 27.9% sensitivity for full-thickness tears, underscoring its strength as a rule-in rather than rule-out sign [13].

It is also interesting to contextualize our findings with other diagnostic tools. Ultrasound is a popular modality for rotator cuff tear detection, boasting high accuracy in experienced hands. Ultrasound data likewise confirm that full-thickness supraspinatus tears show a significantly smaller AHI than partial tears or intact cuffs, with a clear severity correlation [14]. However, ultrasound can be operator-dependent. A plain radiograph showing a high-riding humeral head is an objective sign that is easy to detect and can complement ultrasound findings. Additionally, the radiograph can reveal other info like calcific tendinitis or arthritic changes.

Limitations

We acknowledge several limitations in our study. The sample size (n=90) is moderate and confined to a single institution with a short enrollment period; while our power was sufficient to detect differences in AHI, a larger sample might allow more precise estimates of sensitivity and better generalization.

Another limitation is that our radiographic measurements were done on chest radiographs rather than dedicated shoulder AP views in neutral rotation. While the chest X-ray AP includes the shoulder, it is not tailored for evaluating subacromial space like an AP shoulder (Grashey view) would be. Interestingly, acromiohumeral distance at 90° abduction remained larger in asymptomatic than symptomatic tear shoulders, suggesting preserved dynamic clearance may modulate symptom expression [15]. In most cases, this was acceptable, but subtle differences in arm rotation could affect AHI by a millimeter or so. In future studies, standardized shoulder radiographs might be preferable. However, since many rotator cuff tear patients do get chest X-ray initially, our findings remain applicable. We also did not specifically analyze acromial index or critical shoulder angle - other radiographic indices that have been linked to cuff tear risk - as our focus was AHI. And we did not perform arthroscopic confirmation in all cases. Nonetheless, MRI is an accepted reference with a sensitivity of ~90% for rotator cuff tears.

Future directions

Further research could investigate if measurement of AHI in different arm positions (such as an AP radiograph with arm actively abducted, sometimes called an “active abduction view”) increases sensitivity for detecting tears - acute tears might show dynamic upward migration only when the arm is raised. Additionally, exploring the role of AHI in post-operative scenarios (e.g., does an increased AHI post rotator cuff repair correlate with healing) could be valuable, as a persistently narrow AHI after repair might indicate risk of re-tear.

## Conclusions

In conclusion, our study demonstrates that the acromiohumeral interval on plain radiographs is a useful diagnostic marker for rotator cuff tears with high specificity. A significantly narrowed AHI on a routine chest or shoulder X-ray should alert clinicians to the high likelihood of a full-thickness rotator cuff tear and possibly extensive tendon damage. Conversely, a normal AHI cannot alone rule out a tear, particularly a partial tear, so clinical judgment and further imaging remain necessary for comprehensive evaluation. Plain radiography, with attention to the AHI, provides an important piece of the diagnostic imaging in rotator cuff disease and can guide subsequent management. In practical terms, radiologists should routinely assess the acromiohumeral interval on shoulder or chest X-rays in patients with shoulder pain. Reporting a narrowed AHI and its implication can assist referring clinicians in recognizing a likely rotator cuff tear.

Hence, the acromiohumeral interval on plain radiographs is a simple yet powerful metric in the evaluation of rotator cuff pathology. Incorporating this measurement into routine radiographic analysis enhances the detection of significant rotator cuff injuries and provides insight into the chronicity and severity of tears. This radiographic sign, in conjunction with clinical assessment and confirmatory MRI, can improve diagnostic confidence and inform management decisions in patients with rotator cuff disease.
